# The use of rehabilitation among patients with breast cancer: a retrospective longitudinal cohort study

**DOI:** 10.1186/1472-6963-12-282

**Published:** 2012-08-28

**Authors:** Yi-Hsien Lin, Po-Jung Pan

**Affiliations:** 1 School of Medicine, National Yang-Ming University, Taipei, Taiwan; 2 Division of Radiotherapy, Cheng Hsin General Hospital, Taipei, Taiwan; 3 Department of Physical Medicine & Rehabilitation, National Yang-Ming University Hospital, 260 Xin-Min Road No. 152, I-Lan, Taiwan

**Keywords:** Breast cancer, Rehabilitation, Physical therapy, Health insurance

## Abstract

**Background:**

Breast cancer is the most common malignancy in women. Along with improvements in treatment, the number of women who survive breast cancer has increased. Rehabilitation can alleviate post-treatment side effects and maintain quality of life. This study aimed to explore the use of rehabilitation among a cohort of patients diagnosed with breast cancer.

**Methods:**

A retrospective longitudinal cohort study was conducted using a National Health Insurance (NHI) research database in Taiwan. The study cohort consisted of 632 patients with breast cancer diagnosed in 2005. Their NHI claims over a period spanning 2005 through 2009 were analyzed.

**Results:**

Overall, 39.6% of the cohort received rehabilitation therapy, with 9,691 rehabilitation visits claimed (an average of 38.8 visits per user). The prevalence of rehabilitation service use among the cohort was 16.5%, 13.3%, 13.0%, 13.3%, and 12.8% in the years 2005 through 2009, respectively. The average number of visits per rehabilitation user was 16.8, 25.0, 31.1, 24.2, and 23.8 in the years 2005 through 2009, respectively. Most rehabilitation therapy occurred as an outpatient service (96.0%). Physical therapy was the most commonly used form of rehabilitation (84.2%), followed by occupational therapy (15.4%). The most frequently recorded diagnoses were malignant neoplasm of the female breast, peripheral enthesopathies and allied syndromes, and osteoarthrosis and allied disorders.

**Conclusions:**

Only a small proportion of patients with breast cancer received rehabilitation therapy in the first five years after diagnosis. The average number of rehabilitation visits per user peaked in the third year after diagnosis.

## Background

Breast cancer is the most common malignancy in women, with approximately 1.38 million new patients and 459,000 deaths per year worldwide [1]. Depending on breast cancer stage and characteristics, treatment may include surgery, radiotherapy, chemotherapy and/or hormonal and target therapy [2,3]. With screening and treatment strategy advance, the 5-year survival of patients detected with early stage breast cancer is between 80% and 90% [3,4]. Therefore, an increasing number of women survive breast cancer. The concept of cancer rehabilitation is not new; however, it is gaining increasing recognition [5-7]. Survivors of breast cancer must cope with the consequences of their medical treatment. Local problems (such as pain, lymphedema, and shoulder dysfunction) and systemic problems (such as neuropathy, hormone disorder, and psychological problems) may occur after diagnosis and treatment, and ongoing treatment of these conditions may be necessary [8-10]. These ongoing problems faced by the patients increase the need for rehabilitation. Several studies have shown that rehabilitation can alleviate post-treatment side effects, maintain quality of life, and improve the survival [11-15]. However, information on rehabilitation service use among patients with breast cancer remains scarce.

Taiwan launched a National Health Insurance (NHI) program in 1995. More than 99% of the 23 million Taiwanese citizens and legal residents are enrolled. The range of care covered by NHI includes inpatient and ambulatory care, dental services, traditional Chinese medicine, child delivery services, rehabilitation, home care, and chronic mental illness care. Rehabilitation, which is mainly used for physical conditions, comprises physical, occupational, and speech therapies. The nutrition and dietetic services of cancer are neither included as rehabilitation nor covered by NHI. There are various rehabilitation therapy categories listed by NHI. According to treatment content and program duration, the cost of an insurance claim for these services fits into one of several different degrees. In physical therapy, there are five claim degrees—simple, simple-moderate, moderate-moderate, moderate-complicated, and complicated. Occupational therapy claim degrees are similar to those of physical therapy, but without the simple-moderate degree. Speech therapy has only three claim degrees. Taking duration of physical therapy as an example, moderate and moderate-complicated degree claims correspond with physical therapy program durations of at least 30 and 50 min, respectively. More than 90% of medical providers have a contract with the Bureau of NHI. The National Health Insurance Research Database (NHIRD) was introduced for research purposes. In this study, we use the NHIRD to explore the prevalence, patterns, and costs of rehabilitation for patients with breast cancer.

Using the NHIRD, we conducted a retrospective longitudinal study of rehabilitation use among a cohort of patients with breast cancer. We examined the characteristics and trends of rehabilitation use among this group of patients.

## Methods

### Data sources

This retrospective longitudinal study used the Longitudinal Health Insurance Database 2005 (LHID2005), which was obtained from the NHIRD. LHID2005 contains all original claim data of 1 million people randomly sampled from the 23 million beneficiaries in the NHIRD. No significant differences exist in the age, gender, and insured amount distributions between patients in the LHID2005 and the original NHIRD. The patient identity and institution data in the NHIRD were cryptographically scrambled by NHI before being made available to researchers. The study was approved by The Institutional Review Board of National Yang-Ming University Hospital.

### Study samples

Patients with breast cancer were identified in the registry as patients with a catastrophic illness with International Classification of Diseases, Ninth Revision, Clinical Modification (ICD-9-CM) code 174.xx. The study cohort consisted of 632 patients who were registered with a diagnosis of breast cancer in 2005. Patients in the cohort were divided into two subgroups; those who had used rehabilitation services and those who had not. Their rehabilitation service visits in 2005 through 2009 listed in the inpatient and outpatient claims file were analyzed retrospectively.

### Costs

All costs were direct medical costs and are presented in U.S. dollars (US$1 = NT$32.42 based on the average exchange rate of the 2005–2009 period). Only insurance-covered services were included. The costs in this study included medical benefit claims and copayments. Patient copayments excluded registration fees. In this study, rehabilitation costs included those for physical therapy, occupational therapy, speech/swallowing therapy, evaluation, and splint fees.

### Statistics

Data were processed and analyzed using SPSS for Windows Version 13.0 (SPSS Inc, Chicago, IL, USA). The frequency distribution for each variable was examined using chi-squared tests. A *p*-value < 0.05 was considered statistically significant.

## Results

In the cohort, 250 (39.6%) patients with breast cancer used insurance-covered rehabilitation at least once in 2005 through 2009.

### Patient demographics

Patient demographics are presented in Table 1. The median age was 51.5 in rehabilitation nonusers, and 52.8 in rehabilitation users. Proportionately, there were slightly more rehabilitation users than nonusers in their 40s or 60s. For income (insured payroll-related amounts), there were slightly more rehabilitation users than nonusers with low (monthly amount US$1 to US$605) or middle income (monthly amount US$606 to US$1,210). Regarding insured regions, there were slightly more rehabilitation users than nonusers in southern and eastern Taiwan. For insured unit (the occupational or identity category in which the ensured person is enrolled), there were more rehabilitation users than nonusers who were members of occupational unions and foreign crew, farmers, fishermen, low-income households, veterans, and other regional populations. The overall differences in age, insured amount, insured region, and insured unit between rehabilitation nonusers and users were not significant.

**Table 1:** Patient demographics

**Characteristics**	**Rehabilitation non-users**		**Rehabilitation users**		***p*value**^**d**^
	**Total**	**%**	**Total**	**%**	
**No. of patients**	382		250		
**Age**					0.397
Median (years)	51.5		52.8		
< 40	39	10.2	21	8.4	
40’s	118	30.9	81	32.4	
50’s	136	35.6	77	30.8	
60’s	54	14.1	48	19.2	
> 70	35	9.2	23	9.2	
**Insured amount**^a^**(US$/month)**					0.645
Total	103	27.0	60	24.0	
Low income (1–605)	152	39.8	110	44.0	
Middle income (606–1210)	80	20.9	54	21.6	
High income (> 1210)	47	12.3	26	10.4	
**Insured region**					0.713
Northern Taiwan	208	54.5	134	53.6	
Central Taiwan	63	16.5	35	14.0	
Southern Taiwan	102	26.7	73	29.2	
Eastern Taiwan & Offshore islands	9	2.4	8	3.2	
**Insured unit**					0.713
Employees of government, school, enterprises or institutions	108	28.3	61	24.4	
Members of occupational unions & foreign crew	86	22.5	63	25.2	
Farmers & fishermen	44	11.5	34	13.6	
Low-income households	4	1.0	3	1.2	
Veterans & other regional population^b^	37	9.7	29	11.6	
Dependent^c^	103	27.0	60	24.0	

^a^Amount of income on which premiums are levied based on a payroll bracket table.

^b^All family members are unemployed and non-low-income households.

^c^Unemployed or younger than 20 years with an insured family member.

^d^Pearson’s chi-squared test.

### Trends of rehabilitation use

The prevalence of rehabilitation use was 16.5%, 13.3%, 13.0%, 13.3%, and 12.8% in each cross-sectional year of 2005, 2006, 2007, 2008, and 2009, respectively (Table 2). During the 5-year period, 250 rehabilitation users attended 9,691 rehabilitation service visits (an average of 38.8 visits per user). The average number of rehabilitation service visits per user was 16.8, 25.0, 31.1, 24.2, and 23.8 in each cross-sectional year of 2005, 2006, 2007, 2008, and 2009, respectively. The average number of rehabilitation service visits per year reached a peak in the third year after breast cancer diagnosis (2007). The cost of rehabilitation therapy was US$94,337, accounting for 0.9% of total medical cost. The average cost per user was $178.00, $257.10, $319.10, $207.90, and $205.00 in each cross-sectional year of 2005, 2006, 2007, 2008, and 2009, respectively. The average cost per user during the study period was US$377.30.

**Table 2:** Trends of rehabilitation use among patients with breast cancer

**Year**	**All medical users**	**Rehabilitation users**	**Percentage of all medical users**	**Rehabilitation therapy visits**	**Average visits per user**	**Cost (US$)**	**Percentage of all cost**	**Average cost per user**
2005	632	104	16.5%	1743	16.8	18512	0.5%	178.0
2006	616	82	13.3%	2050	25.0	21085	0.9%	257.1
2007	591	77	13.0%	2392	31.1	24594	1.6%	319.4
2008	566	75	13.3%	1813	24.2	15591	1.0%	207.9
2009	555	71	12.8%	1693	23.8	14555	1.0%	205.0
2005–2009	632	250	39.6%	9691	38.8	94337	0.9%	377.3

### Therapy patterns

**Figure 1:**
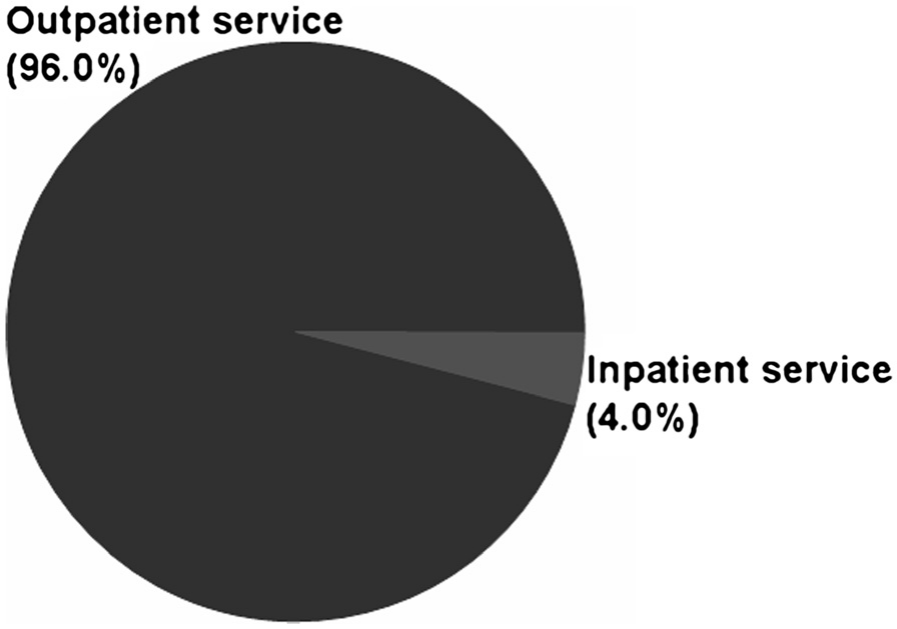
The distribution of rehabilitation use among patients with breast cancer in outpatient and inpatient services from 2005 through 2009 (by prescription).

**Figure 2:**
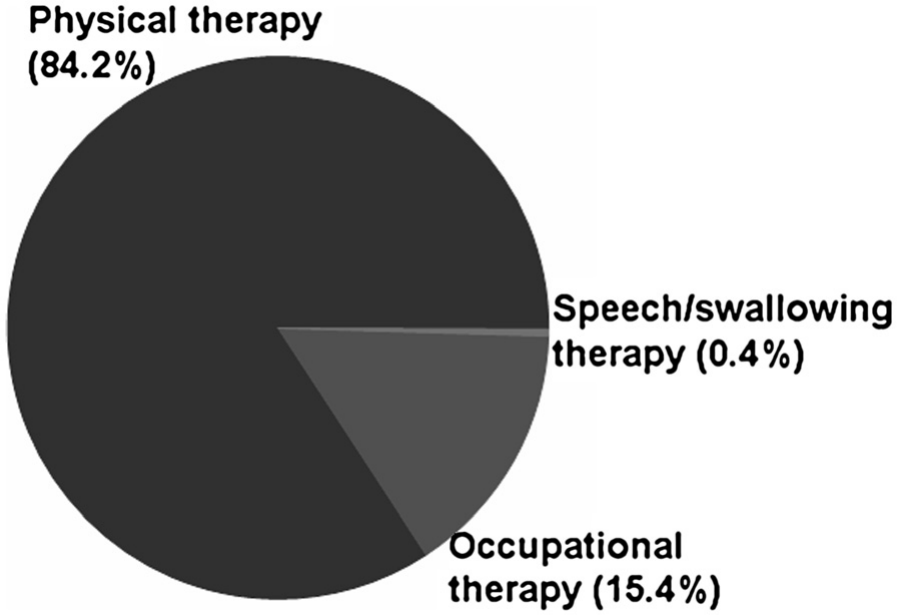
The distribution of rehabilitation use among patients with breast cancer by therapy category, from 2005 through 2009 (by prescription).

Most rehabilitation therapy occurred as an outpatient service (96.0%) (Figure 1). Concerning therapy categories, physical therapy (84.2%) was the most commonly used, followed by occupational therapy (15.4%) and speech/swallowing therapy (0.4%) (Figure 2). Physical therapy moderate-moderate degree (60.5%), physical therapy moderate-complicated degree (16.2%), and occupational therapy moderate degree (6.5%) were the most commonly used programs (Table 3).

**Table 3:** Rehabilitation therapy category distribution among patients with breast cancer

**Therapy**	**No. of prescriptions**	**%**
Physical Therapy -- Moderate-moderate	5861	60.5
Physical Therapy -- Moderate-complicated	1573	16.2
Occupational Therapy -- Moderate	631	6.5
Physical Therapy -- Complicated	550	5.7
Occupational Therapy -- Complicated	393	4.1
Occupational Therapy -- Moderate-complicated	220	2.3
Occupational Therapy -- Simple	203	2.1
Physical Therapy -- Simple	157	1.6
Speech & swallowing Therapy -- Complicated	36	0.4
Physical Therapy Evaluation	20	0.2
Spasticity reduction splint	9	0.1
Spasticity reduction splint (Material fee)	9	0.1
Occupational Therapy Evaluation	7	0.1
Resting splint, short leg	7	0.1
Resting splint, short leg (Material fee)	7	0.1
Speech & swallowing Therapy Evaluation	4	0.0
Cock-up, splint	2	0.0
Cock-up, splint (Material fee)	2	0.0
Total	9691	100.0

### Diagnoses of rehabilitation visits

According to NHI regulation, diagnosis codes were recorded in ICD-9-CM format. Up to three diagnosis codes were in each outpatient prescription, and up to five diagnosis codes were in each inpatient prescription. The most frequently recorded diagnosis codes were malignant neoplasm of the female breast (2,072), peripheral enthesopathies and allied syndromes (1,232), and osteoarthrosis and allied disorders (999) (Table 4).

**Table 4:** Top ten diagnoses among rehabilitation users in this cohort

**ICD-9-CM code**	**Disease**	**No. of prescriptions**
174	Malignant neoplasm of female breast	2072
726	Peripheral enthesopathies and allied syndromes	1232
715	Osteoarthrosis and allied disorders	999
434	Occlusion of cerebral arteries	924
721	Spondylosis and allied disorders	856
729	Other disorders of soft tissues	823
457	Noninfectious disorders of lymphatic channels	631
438	Late effects of cerebrovascular disease	597
724	Other and unspecified disorders of back	432
437	Other and ill-defined cerebrovascular disease	370

ICD-9-CM: International Statistical Classification of Disease and Related Health Problems, Ninth Revision, Clinical Modification.

## Discussion

Currently, most studies on the use of rehabilitation therapies among patients with breast cancer are cross-sectional. This is the first longitudinal cohort study to report on rehabilitation use among patients with breast cancer. Numerous follow-up studies are limited by low response and high dropout rates. The NHIRD covers over 90% of residents and medical institutes in Taiwan. It tracks each insured patient’s claims over time. In this study, all claims from different medical institutes during the study period were obtained for analysis. This was accomplished to circumvent the limitation of patient dropout that is present in most longitudinal studies. Furthermore, the measurement of rehabilitation use was based on insurance claims to avoid recall bias.

Previous studies have found that at 5-year follow-up, shoulder or arm pain occurred in 30% to 40% of patients, restricted shoulder mobility occurred in 15% to 30%, and lymphedema occurred in 10% to 40%, depending on the method of assessment [16-19]. In this study, the prevalence of rehabilitation use among patients with breast cancer was 12.8% to 16.5% in the first 5 years after diagnosis (Table 2), which is substantially lower than the rate of complications after breast cancer treatment [8]. In contrast to various social security and healthcare systems in different countries [20], the cancer rehabilitation services of NHI in Taiwan are cheaper and more convenient for enrolled residents. However, the uptake of rehabilitation remains low. There were no significant differences in age, insured amount, insured region, or insured unit between rehabilitation service nonusers and users (Table 1). Therefore, the factors influencing the use of rehabilitation among survivors of breast cancer do not include age, income, location, or occupation/identity. In the first few years after diagnosis, patients with breast cancer must spend a considerable amount of time caring for their wounds and receiving radiotherapy or chemotherapy [21]. Other problems, such as post-treatment fatigue and psychosocial disturbance also interfere with the survivor’s ability to seek rehabilitation services [8]. Mobility problems, such as the sequelae of a stroke in patients who develop breast cancer, are examples of physical barriers that prevent patients from participating in rehabilitation programs [22]. Many of these barriers represent perfect indications for referral to rehabilitation services.

Survivors of breast cancer who seek alternative or complementary therapies to relieve their discomfort decrease their use of rehabilitation [23,24]. In Taiwan, breast cancer survivors can participate the mutual aid group (mutual support and self-help group), which was mainly composed of patients, physicians (oncologists or surgeons), and social workers. The group provides support via the specialists or the patients themselves. It is free for cancer survivors or their families to participate in mutual aid group activities or lectures. Through encouragement and support, mutual aid groups may indirectly increase the number of patients with breast cancer seeking rehabilitation services. The education and information provided by clinicians to survivors of breast cancer are seemingly inadequate [25].

Table 2 shows that the largest number of patients used rehabilitation therapy during the first year after breast cancer diagnosis. This is foreseeable, because patients need more help and information about the disease immediately after their diagnosis. In contrast, the average number of rehabilitation visits per year peaked in the third year after diagnosis. This may be related to exacerbated lymphedema symptoms after surgery or radiotherapy in the first 3 years [8,18,26]. The importance of early diagnosis and treatment of small-volume lymphedema, especially in the first year after breast cancer operation was recently proposed [27-29]. In the early stage of lymphedema, the first symptoms are subjective sensations of tightness or palpable tightness in the subcutaneous depth of the affected arm. These symptoms may be reversed by means of conservative treatments including compression bandaging, wearing a sleeve, lymphatic drainage massage, and pneumatic pumping. Without treatment, edema volume will increase, pain will exacerbate, and the fat tissue may eventually develop fibrosis. This is considered chronic lymphedema, and is often irreversible. According to the results of this study, patients with breast cancer delayed their use of rehabilitation. Therefore, more emphasis should be placed on the golden intervention time, arm lymphedema diagnosed within12 months after operation, for rehabilitation visits [27].

Frequently, a rehabilitation program for survivors of breast cancer must address multiple problems such as pain, edema, and limited shoulder mobility. The duration of each appointment is often over 30 minutes, which can be attributed to the moderate-moderate or moderate-complicated claim degrees. In addition, manual techniques (for example, complex decongestive therapy or lymph drainage massage) must be performed by the physical therapist on a one-on-one basis [30]. This is compatible with the high percentage of moderate-moderate degree physical therapy claims (Table 3). Meanwhile, the complications experienced by survivors of breast cancer often occur months or years after diagnosis and persist over time. Most of the late effects can be treated through outpatient services (Figure 1).

Survivors of breast cancer visit their medical service providers not only because of complications from breast cancer, but also for general rehabilitation services. Analyzing the diagnoses among users of rehabilitation can reveal co-morbidity with other diseases (Table 4). In addition to physical therapy, survivors of breast cancer occasionally need help from occupational or speech therapists (Figure 2). This is a reminder that rehabilitation services for survivors of breast cancer should be multidimensional, especially for older patients or people who have suffered other disabilities, such as a stroke.

Several limitations of this study must be acknowledged. The NHIRD is primarily used for administrative purposes. The clinical characteristics of breast cancer among the survivors were not included in this study [31]. Further research with detailed clinical stage and treatment methods should be designed for an analysis of rehabilitation use. In addition, psychosocial therapy is an important part of multidisciplinary rehabilitation programs. However, insurance claims for codes related to psychosocial therapy are processed by the Psychology Department in Taiwan, rather than the Department of Physical Medicine and Rehabilitation. Use of psychosocial consultations and therapy was not counted in this study. Moreover, the date of diagnosis was defined as the date patients with breast cancer were included in the registry for patients with catastrophic illness. The time lag between diagnosis and registry might be up to several months. For example, a patient diagnosed with breast cancer in late 2005 would have a shorter period to seek rehabilitation therapy during that year. Therefore, the use of rehabilitation may be slightly underestimated in the first year (Table 2).

## Conclusions

Only small proportion of patients with breast cancer received rehabilitation services in the first five years after diagnosis. The average number of rehabilitation visits per user peaked in the third year after diagnosis. Health care providers should recognize the needs of patients with breast cancer and provide timely information about rehabilitation therapy to relieve their symptoms.

## Competing interests

The authors declare that they have no competing interests.

## Authors’ contributions

YHL and PJP contributed to study design, journal review, and manuscript preparation. YHL performed the statistical analysis. PJP revised the manuscript. Both authors read and approved the final manuscript.

## Pre-publication history

The pre-publication history for this paper can be accessed here:

http://www.biomedcentral.com/1472-6963/12/282/prepub

## References

[B1] Globocan.iarc.fr. Lyon: Globocan 2008 http://globocan.iarc.fr/

[B2] Al-Benna S Poggemann K Steinau HU Steinstraesser L Diagnosis and management of primary breast sarcoma Breast Cancer Res Treat 2010 122 619 626 10.1007/s10549-010-0915-y 20480227

[B3] Maughan KL Lutterbie MA Ham PS Treatment of breast cancer Am Fam Physician 2010 81 1339 1346 20521754

[B4] National Cancer Institute Surveillance Epidemiology and End Results: Cancer Statistics[http://seer.cancer.gov/statistics/]

[B5] National Cancer Action Team Rehabitation Care Pathway - Breast http://ncat.nhs.uk/sites/default/files/NCAT_Rehab_Breast.pdf

[B6] Harris SR Schmitz KH Campbell KL McNeely ML Clinical practice guidelines for breast cancer rehabilitation: syntheses of guideline recommendations and qualitative appraisals Cancer 2012 118 Suppl 8 2312 2324 22488705 10.1002/cncr.27461

[B7] Gilchrist LS Galantino ML Wampler M Marchese VG Morris GS Ness KK A framework for assessment in oncology rehabilitation Phys Ther 2009 89 286 306 10.2522/ptj.20070309 19147708 PMC2967778

[B8] Ewertz M Jensen AB Late effects of breast cancer treatment and potentials for rehabilitation Acta Oncol 2011 50 187 193 10.3109/0284186X.2010.533190 21231780

[B9] Chalasani P Downey L Stopeck AT Caring for the breast cancer survivor: a guide for primary care physicians Am J Med 2010 123 489 495 10.1016/j.amjmed.2009.09.042 20569749

[B10] Grunfeld E Dhesy-Thind S Levine M Steering Committee on Clinical Practice Guidelines for the Care and Treatment of Breast Cancer. Clinical practice guidelines for the care and treatment of breast cancer: follow-up after treatment for breast cancer (summary of the 2005 update) CMAJ 2005 172 1319 1320 10.1503/cmaj.045062 15883407 PMC557103

[B11] Fernández-Lao C Cantarero-Villanueva I Fernández-de-Las-Peñas C Del Moral-Ávila R Castro-Sánchez AM Arroyo-Morales M Effectiveness of a Multidimensional Physical Therapy Program on Pain, Pressure Hypersensitivity, and Trigger Points in Breast Cancer Survivors: A Randomized Controlled Clinical Trial Clin J Pain 2011 [Epub ahead of print] 10.1097/AJP.0b013e318225dc02 21705873

[B12] Poorkiani M Abbaszadeh A Hazrati M Jafari P Sadeghi M Mohammadianpanah M The effect of rehabilitation on quality of life in female breast cancer survivors in Iran Indian J Med Paediatr Oncol 2010 31 105 109 10.4103/0971-5851.76190 21584214 PMC3089917

[B13] Gupta AD Lewis S Shute R Patients living with cancer - the role of rehabilitation Aust Fam Physician 2010 39 844 846 21301657

[B14] Valenti M Porzio G Aielli F Verna L Cannita K Manno R Masedu F Marchetti P Ficorella C Physical exercise and quality of life in breast cancer survivors Int J Med Sci 2008 5 24 28 18219372 10.7150/ijms.5.24 PMC2204041

[B15] Holmes MD Chen WY Feskanich D Kroenke CH Colditz GA Physical activity and survival after breast cancer diagnosis JAMA 2005 293 2479 2486 10.1001/jama.293.20.2479 15914748

[B16] Nesvold IL Reinertsen KV Fosså SD Dahl AA The relation between arm/shoulder problems and quality of life in breast cancer survivors: a cross-sectional and longitudinal study J Cancer Surviv 2011 5 62 72 10.1007/s11764-010-0156-4 20972640 PMC3040353

[B17] Sagen A Kåresen R Risberg MA Changes in arm morbidities and health-related quality of life after breast cancer surgery—a five year follow-up study Acta Oncol 2009 48 1102 1110 10.3109/02841860903061683 19863218

[B18] Hopwood P Haviland JS Sumo G Mills J Bliss J Yarnold J START Trial Management Group START Trial Management Group Comparison of patient-reported breast, arm, and shoulder symptoms and body image after radiotherapy for early breast cancer: 5-year follow-up in the randomized Standardisation of Breast Radiotherapy (START) trials Lancet Oncol 2010 11 231 240 10.1016/S1470-2045(09)70382-1 20138809

[B19] Tsai RJ Dennis LK Lynch CF Snetselaar LG Zamba GKD Scott-Conner C The risk of developing arm lymphedema among breast cancer survivors: A meta-analysis of treatment factors Ann Surg Oncol 2009 16 1959 1972 10.1245/s10434-009-0452-2 19365624

[B20] Hellbom M Bergelt C Bergenmar M Gijsen B Loge JH Rautalahti M Smaradottir A Johansen C Cancer rehabilitation: A Nordic and European perspective Acta Oncol 2011 50 179 186 10.3109/0284186X.2010.533194 21231779

[B21] Cheville AL Tchou J Barriers to rehabilitation following surgery for primary breast cancer J Surg Oncol 2007 95 409 418 10.1002/jso.20782 17457830

[B22] Iezzoni LI Kilbridge K Park ER Physical access barriers to care for diagnosis and treatment of breast cancer among women with mobility impairments Oncol Nurs Forum 2010 37 711 717 10.1188/10.ONF.711-717 21059583 PMC3008578

[B23] Lin YH Chen KK Chiu JH Prevalence, patterns, and costs of Chinese medicine use among patients with breast cancer: a population-based study in Taiwan Integrative cancer therapies 2010 9 16 23 10.1177/1534735409359073 20308084

[B24] Gerson-Cwilich R Serrano-Olvera A Villalobos-Prieto A Complementary and alternative medicine (CAM) in Mexican patients with cancer Clin Transl Oncol 2006 8 200 207 10.1007/s12094-006-0011-2 16648120

[B25] Binkley JM Harris SR Levangie PK Pearl M Guglielmino J Kraus V Rowden D Patient perspectives on breast cancer treatment side effects and the prospective surveillance model for physical rehabilitation for women with breast cancer Cancer 2012 118 Suppl 8 2207 2216 22488695 10.1002/cncr.27469

[B26] Petrek JA Senie RT Peters M Rosen PP Lymphedema in a cohort of breast carcinoma survivors 20 years after diagnosis Cancer 2001 92 1368 1377 10.1002/1097-0142(20010915)92:6<1368::AID-CNCR1459>3.0.CO;2-9 11745212

[B27] Johansson K Branje E Arm lymphoedema in a cohort of breast cancer survivors 10 years after diagnosis Acta Oncol 2010 49 166 173 10.3109/02841860903483676 20100154

[B28] Torres Lacomba M Yuste Sánchez MJ Zapico Goñi A Prieto Merino D Mayoral del Moral O Cerezo Téllez E Minayo Mogollón E Effectiveness of early physiotherapy to prevent lymphoedema after surgery for breast cancer: randomised, single blinded, clinical trial BMJ 2010 12 340 5396 10.1136/bmj.b5396 PMC2806631 20068255

[B29] Stout Gergich NL Pfalzer LA McGarvey C Springer B Gerber LH Soballe P Preoperative assessment enables the early diagnosis and successful treatment of lymphedema Cancer 2008 112 2809 2819 10.1002/cncr.23494 18428212

[B30] Cheifetz O Haley L Breast Cancer Action. Management of secondary lymphedema related to breast cancer Can Fam Physician 2010 56 1277 1284 21375063 PMC3001918

[B31] Cheville AL Kornblith AB Basford JR An examination of the causes for the underutilization of rehabilitation services among people with advanced cancer Am J Phys Med Rehabil 2011 90 Suppl 5 S27 S37 21765261 10.1097/PHM.0b013e31820be3be

